# Sensory Impairment and Social Isolation: Implications for the Hispanic Population

**DOI:** 10.1093/geroni/igaa057.2096

**Published:** 2020-12-16

**Authors:** Stephanie Richardson, Corinna Tanner, Jeremy Yorgason

**Affiliations:** 1 Brigham Young University, Provo, Utah, United States; 2 Brigham Young University, provo, Utah, United States

## Abstract

Although the likelihood of developing a disability increases with age among all demographics, older adults of hispanic origin are more likely to experience vision and hearing impairment than both their white and black non-hispanic counterparts. Both hearing impairment and vision impairment are known risk factors for social isolation, yet little research has examined this association in Hispanic populations. Using data from 472 Hispanic and 5,186 White participants of the NHATS study, we examined 8-year trajectories of social isolation, along with how sensory impairment was associated with initial levels and change over time. Findings suggest that sensory impairments are linked with steeper increases over time among White participants. Among Hispanics vision and hearing impairments were linked with higher initial levels of social isolation, yet no associations were found across time. It may be that Hispanic older adults maintain social connections across time despite potentially isolating sensory impairments.

